# Targeting Sphingolipids for Cancer Therapy

**DOI:** 10.3389/fonc.2021.745092

**Published:** 2021-10-19

**Authors:** Osmel Companioni, Cristina Mir, Yoelsis Garcia-Mayea, Matilde E. LLeonart

**Affiliations:** ^1^ Biomedical Research in Cancer Stem Cells Group, Vall d’Hebron Research Institute (VHIR), Universitat Autònoma de Barcelona, Barcelona, Spain; ^2^ Spanish Biomedical Research Network Center in Oncology, CIBERONC, Madrid, Spain

**Keywords:** cancer, sphingolipids, preclinical, clinical studies, therapy

## Abstract

Sphingolipids are an extensive class of lipids with different functions in the cell, ranging from proliferation to cell death. Sphingolipids are modified in multiple cancers and are responsible for tumor proliferation, progression, and metastasis. Several inhibitors or activators of sphingolipid signaling, such as fenretinide, safingol, ABC294640, ceramide nanoliposomes (CNLs), SKI-II, α-galactosylceramide, fingolimod, and sonepcizumab, have been described. The objective of this review was to analyze the results from preclinical and clinical trials of these drugs for the treatment of cancer. Sphingolipid-targeting drugs have been tested alone or in combination with chemotherapy, exhibiting antitumor activity alone and in synergism with chemotherapy *in vitro* and *in vivo*. As a consequence of treatments, the most frequent mechanism of cell death is apoptosis, followed by autophagy. Aslthough all these drugs have produced good results in preclinical studies of multiple cancers, the outcomes of clinical trials have not been similar. The most effective drugs are fenretinide and α-galactosylceramide (α-GalCer). In contrast, minor adverse effects restricted to a few subjects and hepatic toxicity have been observed in clinical trials of ABC294640 and safingol, respectively. In the case of CNLs, SKI-II, fingolimod and sonepcizumab there are some limitations and absence of enough clinical studies to demonstrate a benefit. The effectiveness or lack of a major therapeutic effect of sphingolipid modulation by some drugs as a cancer therapy and other aspects related to their mechanism of action are discussed in this review.

## Introduction

Sphingolipids are key structural components of cellular membranes containing a backbone of sphingosine (aliphatic amino alcohol) as the base of their structures. They are synthesized, metabolized and trafficked among several cell organelles.​ Sphingolipids are remarkably diverse and have crucial roles in maintaining barrier function and fluidity, as well as regulating the cell cycle, cell motility, differentiation, adhesion, and apoptosis (1).

**Figure 1:**
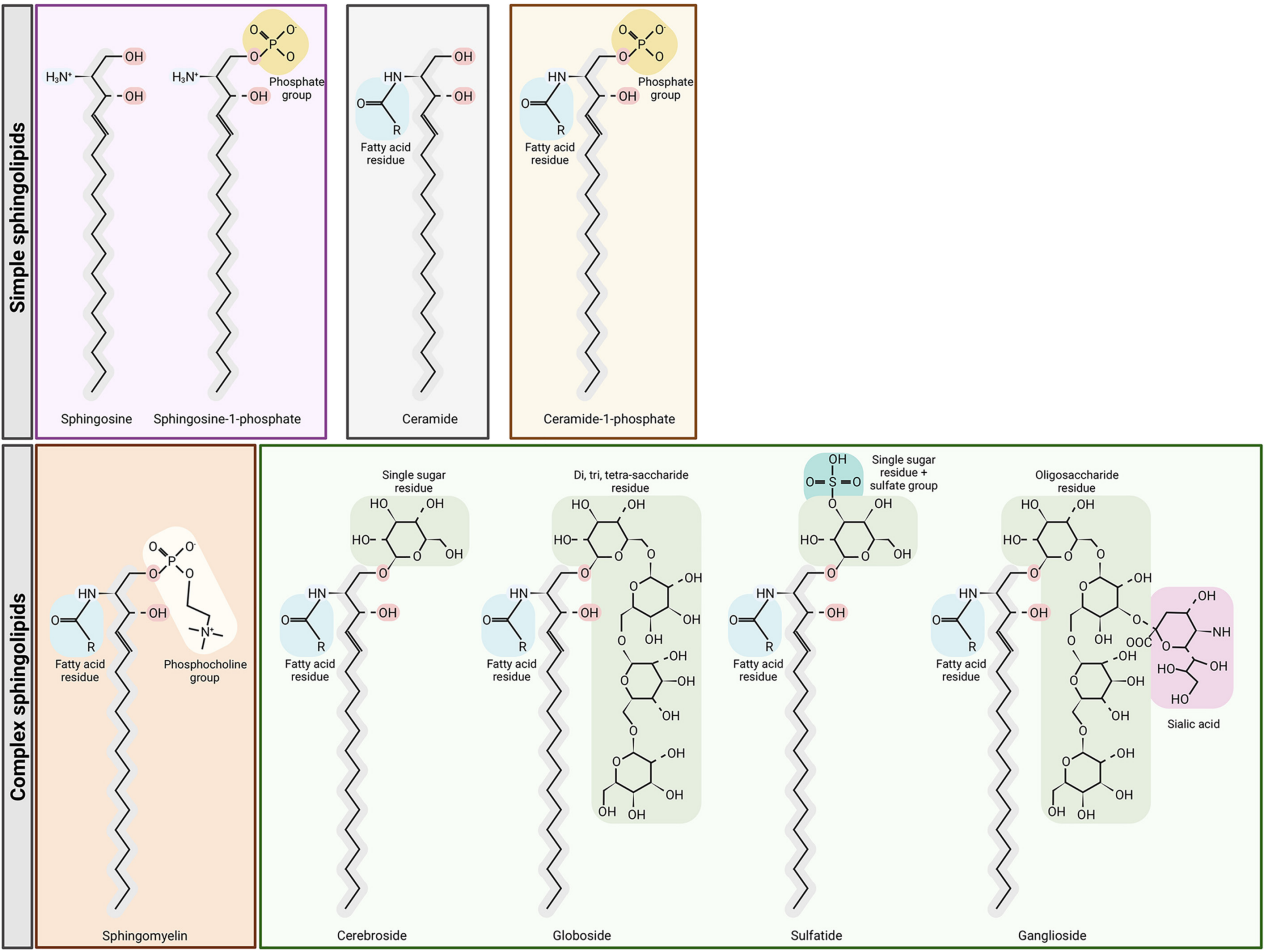
Structures of sphingolipids. Sphingosine is the base for simple sphingolipids. Ceramide contains a fatty acid residue. The addition of a phosphate group to sphingosine or ceramide yields sphingosine-1-phosphate and ceramide-1-phosphate, respectively. Complex sphingolipids are synthesized through ceramide modifications. The addition of a phosphocholine group to ceramide yields sphingomyelin, but the addition of glucose or galactose to ceramide yields glycosphingolipids and sulfatides. Figure created with BioRender.com.

**Figure 2:**
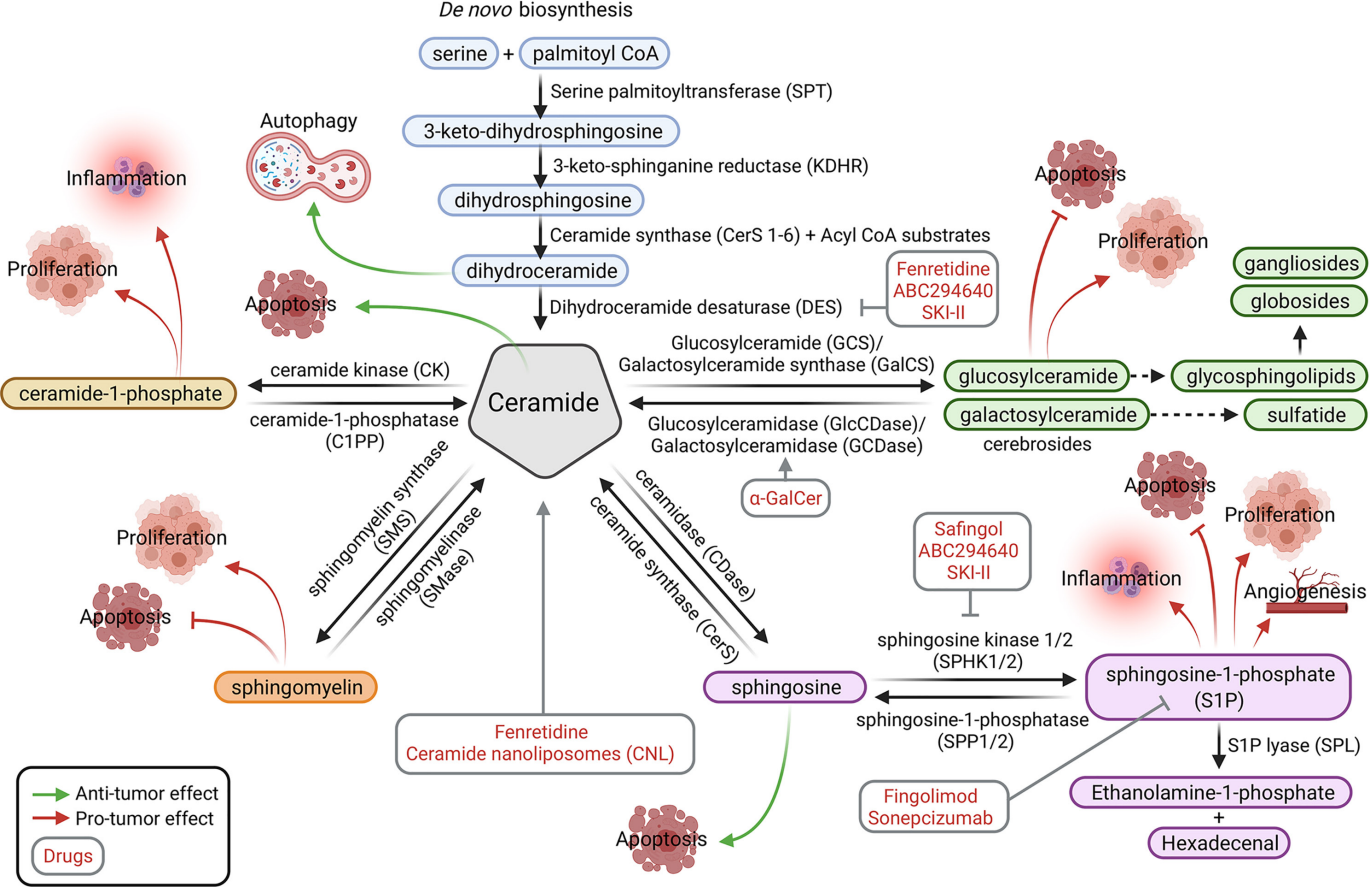
Sphingolipid metabolic pathways. The sphingolipid modulators (in red) and affected tumor processes (processes by which molecules promote tumor growth are indicated with red arrows, whereas those that inhibit tumor growth are indicated with green arrows) are shown. Ceramide is the central molecule that is synthesized through the *de novo* pathway or other catabolic pathways through conversion from ceramide-1-phosphate, sphingomyelin, sphingosine, glycosphingoplidis, or sulfatides (glucosyl or galactosyl-ceramide). Figure created with BioRender.com.

Sphingolipids include ceramides, sphingomyelins, cerebrosides, sulfatides, globosides and gangliosides (**Figure 1**). *De novo* sphingolipid synthesis begins with the formation of 3-keto-dihydrosphingosine by serine palmitoyltransferase (SPT). Next, 3-keto-dihydrosphingosine is reduced to form dihydrosphingosine, which is acylated by a ceramide synthase (CerS) to form dihydroceramide. CerS enzymes have different affinities for acyl-CoA substrates, resulting in the generation of dihydroceramides with differing chain lengths (C14-C26). Dihydroceramides are then desaturated to form ceramides (2, 3). *De novo* generated ceramide is the central hub of the sphingolipid pathway and subsequently has several fates (**Figure 2**). It is phosphorylated by ceramide kinase (CK) to form ceramide-1-phosphate or it can be glycosylated by glucosylceramide synthase to form glycosphingolipids (cerebrosides, globosides, gangliosides). In addition, ceramide can be converted to sulfatides by the action of galactosylceramide synthase followed by cerebroside sulfotransferase (CST). Additionally, ceramide is also converted to sphingomyelin by the addition of a phosphorylcholine headgroup by sphingomyelin synthase (SMS). Finally, ceramide may be degraded by ceramidase (CDase) to form sphingosine. Sphingosine may be phosphorylated by sphingosine kinase 1/2 (SPHK1/SPHK2) to form sphingosine-1-phosphate (S1P), which has a prosurvival role and is critical for immunomodulation (1, 4, 5) (**Figure 2**).

SPHK1/2 are overexpressed in numerous cancer cell types, but catabolic pathways allow the reversion of S1P to ceramide by sphingosine-1-phosphatase (SPP1/2) and ceramide synthase. The complex glycosphingolipids are hydrolyzed to glucosylceramide and galactosylceramide. These lipids are then hydrolyzed by beta-glucosidases and beta-galactosidases (GCDase) to regenerate ceramide. Similarly, sphingomyelin may be degraded by sphingomyelinase (SMase) and ceramide-1-phosphate by ceramide-1-phosphatase (C1PP) to form ceramide (4) (**Figure 2**).

**Figure 3:**
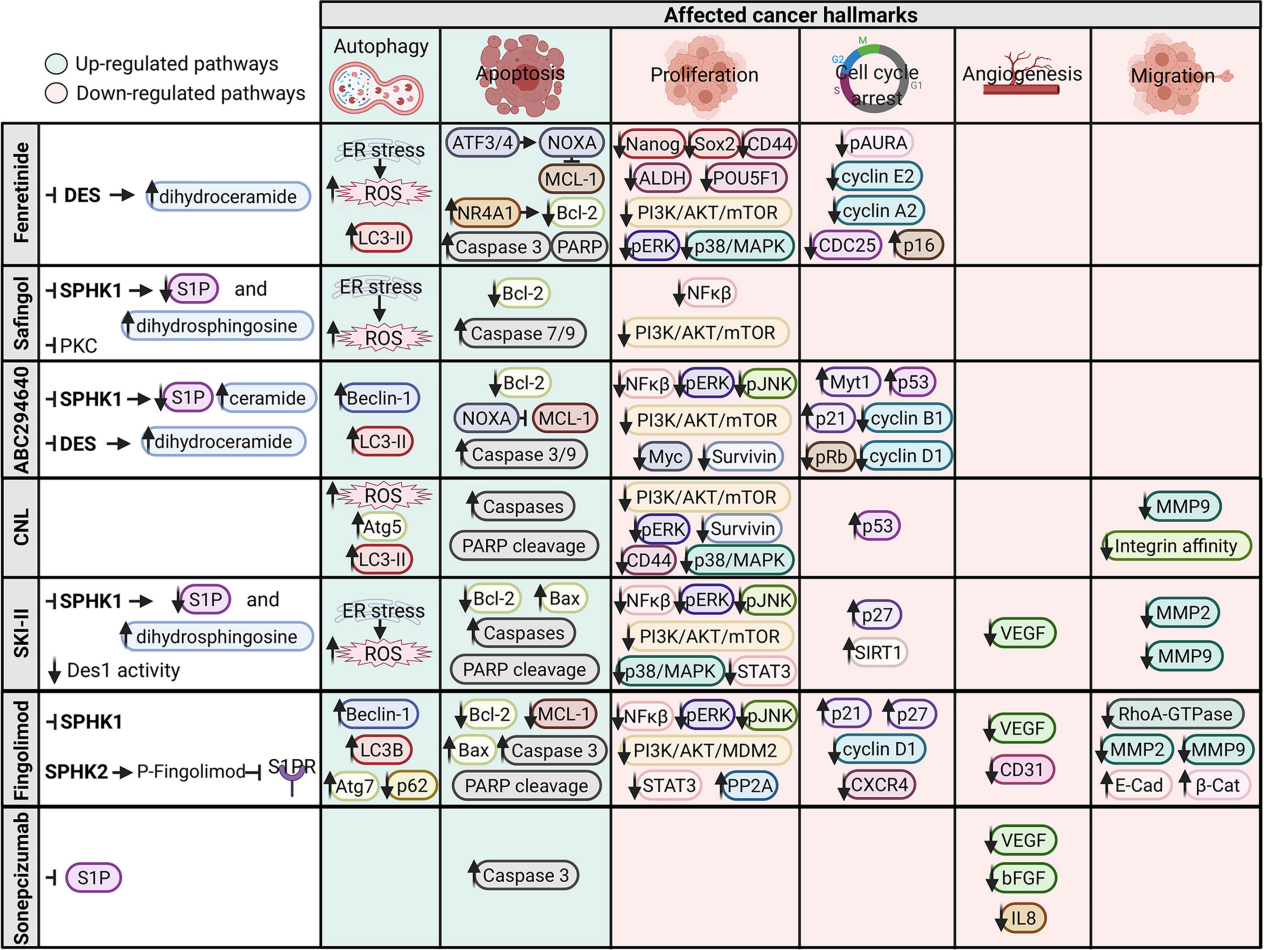
Signaling pathways and cancer hallmarks affected by sphingolipid modulators. Upregulated pathways are indicated in green, and downregulated pathways are indicated in red. Figure created with BioRender.com.

In addition to their roles in the organization of the plasma membrane, sphingolipids also play roles as key molecules in signaling processes [for reviews, see (1, 4)]. A classic example is the increase in ceramide and sphingosine levels caused by chemotherapy, radiation, and/or oxidative stress and the subsequent induction of apoptosis by these molecules. In contrast, sphingosine-1-phosphate displays antiapoptotic and prosurvival properties. Because some of these enzymes regulate the abundance of sphingolipids, their aberrant expression or activity exerts a negative effect on cancer (5). Thus, numerous studies have been performed targeting the enzymes that catabolize ceramide, generate S1P, or regulate sphingolipid levels. Generally, different strategies have been used to exploit the potential antitumor effects of sphingolipids. Among them, we highlight the following biological processes: autophagic cell death, apoptosis induction, including mitochondrial activation (mitophagy), proliferation inhibition, and cell cycle arrest, and effects on angiogenesis and migration (**Figure 3**).

## Chemotherapy and Sphingolipid-Related Drugs

In general, chemotherapy is an effective treatment for cancer due to its ability to kill highly proliferative cells. Chemotherapeutic agents induce stress in cancer cells at the cytoplasmic level (i.e., reactive oxygen species [ROS] production and nuclear DNA damage), and in response, ceramide levels are increased followed by the induction of apoptotic cell death. For example, daunorubicin, etoposide, and gemcitabine have all been described as inducers of *de novo* ceramide generation (6). Chemotherapy resistance has also been linked to altered sphingolipid metabolism, favoring the production of lipid species that ultimately lead to cell survival (7). In this sense, many inhibitors or modulators of sphingolipid metabolism have been developed to kill tumors and reverse chemotherapy resistance (8). These drugs have been employed in preclinical studies using cancer cell lines and orthotopic mouse models, as well as in clinical trials (**Table 1**). In the next sections, this review highlights the drugs most frequently used to target sphingolipid signaling, indicates their mechanisms of action and discusses their successes and limitations in preclinical and clinical trials of cancer treatment. The main results from published preclinical and clinical trials are summarized in **Table 1**.

**Table 1:** Summary of preclinical and clinical studies of inhibitors/modulators of sphingolipids and cancer.

Drug name	Study type	Tumor type
Fenretinide	Preclinical	Lung and colorectal cancer (9)
Fenretinide	Preclinical	Lung, colon and melanoma (10)
Fenretinide	Preclinical	Colon cancer (11)
Fenretinide	Preclinical	AML (12)
Fenretinide	Preclinical	Ovarian and breast cancer (13)
Fenretinide	Preclinical	Myeloid leukemia (14)
Fenretinide	Preclinical	Ovarian cancer (15)
Fenretinide	Preclinical	AML (16)
Fenretinide	Preclinical	AML (17)
Fenretinide	Preclinical	Medulloblastoma (18)
Fenretinide	Preclinical	Liver cancer (19)
Fenretinide	Clinical (phase I), NCT00003191	High-risk solid tumors (20)
Fenretinide	Clinical (phase I), NCT00589381	Solid tumors or lymphoma (21)
Fenretinide	Clinical (phase I)	Breast cancer (22)
Fenretinide	Clinical (phase I)	Hematologic malignancies (23)
Fenretinide	Clinical (phase I), NCT00295919	Neuroblastoma (24)
Fenretinide	Clinical (phase I)	Bladder cancer (25)
Fenretinide	Clinical (phase I-II)	Ovarian cancer (26)
Fenretinide	Clinical (phase I-II)	Breast cancer (27)
Fenretinide	Clinical (phase I-II)	Breast cancer (28)
Fenretinide	Clinical (phase I-II)	Ovarian cancer (29)
Fenretinide	Clinical (phase I-II)	Breast cancer (30)
Fenretinide	Clinical (phase I-II)	Invasive Bladder Cancer (31)
Fenretinide	Clinical (phase II), NCT00077402	Prostate cancer (32)
Fenretinide	Clinical (phase II), NCT00011973	Renal cell carcinoma (33)
Fenretinide	Clinical (phase II), NCT00006080	Recurrent Malignant Glioma (34)
Fenretinide	Clinical (phase II)	Bladder cancer (35)
Fenretinide	Clinical (phase II)	Recurrent prostate cancer (36)
Fenretinide	Clinical (phase II)	Prostate cancer (37)
Fenretinide	Clinical (phase II)	Breast cancer and melanoma (38)
Fenretinide	Clinical (phase II)	Recurrent small cell lung cancer (39)
Fenretinide	Clinical (phase II)	Breast cancer (40)
Fenretinide	Clinical (phase II)	Bladder cancer (31)
Fenretinide	Clinical (phase III), NCT00004154	Bladder Cancer (41)
Fenretinide	Clinical (phase III)	Breast cancer (42)
Fenretinide and ABT-199	Preclinical	Neuroblastoma (43)
Fenretinide and paclitaxel	Preclinical	Ovarian cancer (44)
Fenretinide and lenalidomide	Preclinical	Neuroblastoma (45)
Fenretinide and SAHA	Preclinical	Glioblastoma (46)
Fenretinide and tamoxifen	Clinical (phase I-II)	Breast cancer (47)
Fenretinide and tamoxifen	Clinical (phase I-II)	Breast cancer (48)
Fenretinide and tamoxifen	Clinical (phase I-II)	Breast cancer (49)
Fenretinide and tamoxifen	Clinical (phase II)	Breast cancer (50)
Fenretinide and tamoxifen	Clinical (phase II)	At higher risk for breast cancer (51)
Fenretinide and tamoxifen	Clinical (phase II)	At higher risk for breast cancer (52)
Fenretinide and tamoxifen	Clinical (phase II)	Metastatic breast cancer (53)
Fenretinide and tamoxifen	Clinical (phase II)	Metastatic breast cancer (54)
Fenretinide and tamoxifen	Clinical (phase III), NCT00002646	Receptor-positive breast cancer (55)
Safingol	Preclinical	Isolated hepatocytes (56)
Safingol	Preclinical	Prostate cancer (57)
Safingol	Preclinical	Breast and colon cancer (58)
Safingol	Preclinical	Multiple Myeloma (59)
Safingol	Preclinical	Squamous cell carcinoma (60)
Safingol	Preclinical	Acute myeloid leukemia (61)
Safingol	Preclinical	HNSCC (62)
Safingol	Preclinical	Solid tumors (63)
Safingol	Preclinical	Acute promyelocytic leukemia (64)
Safingol and mitomycin C	Preclinical	Gastric cancer (65)
Safingol and irinotecan	Preclinical	Colon cancer (66)
Safingol and Carboplatin, doxorubicin, gemcitabine, vincristine	Preclinical	Breast, ovarian, lymphoma, mouth cancer (67)
Safingol and (–)-epigallocatechin-O-3-gallate (EGCG)	Preclinical	CLL (68)
Safingol and bortezomib	Preclinical	Triple-negative breast cancer (69)
Safingol and cisplatin	Preclinical	Gastroesophageal cancer (70)
Safingol and cisplatin	Preclinical	HNSCC (71)
Safingol and CNL	Preclinical	AML (72)
Safingol and cysplatin	Clinical (phase I), NCT00084812	Several solid tumors (73)
Safingol and doxorubicin	Clinical (phase I)	Several solid tumors (74)
ABC294640	Preclinical	Breast cancer (75)
ABC294640	Preclinical	Breast cancer (76)
ABC294640	Preclinical	Colon cancer (77)
ABC294640	Preclinical	Breast cancer (78)
ABC294640	Preclinical	Primary effusion lymphoma (79)
ABC294640	Preclinical	Resistant prostate cancer (80)
ABC294640	Preclinical	Prostate cancer (81)
ABC294640	Preclinical	Resistant prostate cancer (82)
ABC294640	Preclinical	Kaposi sarcoma (83)
ABC294640	Preclinical	Skin squamous cell carcinoma (84)
ABC294640	Preclinical	Cervical carcinoma (85)
ABC294640	Preclinical	NSCLC (86)
ABC294640	Preclinical	Epithelial ovarian cancer (87)
ABC294640	Preclinical	Cholangiocarcinoma (88)
ABC294640	Preclinical	Diffuse Glioma (89)
ABC294640	Preclinical	Multiple myeloma (90)
ABC294640	Preclinical	Lymphocyte leukemia (91)
ABC294640	Preclinical	Triple-negative breast cancer (92)
ABC294640	Clinical (phase I), NCT03414489	Several solid tumors (93)
ABC294640 and (SKI)-II	Preclinical	Triple-negative breast cancer (94)
ABC294640 and paclitaxel	Preclinical	Ovarian cancer (95)
ABC294640 and TRAIL	Preclinical	NSCLC (96)
ABC294640, C6 ceramide and SKI-II	Preclinical	Colorectal cancer (97)
ABC294640 and sorafenib	Preclinical	Cholangiocarcinoma (98)
ABC294640 and PDMP	Preclinical	Lung cancer (99)
ABC294640 and SKI	Preclinical	Prostate cancer (100)
ABC294640 and gemcitabine	Preclinical	Pancreatic cancer (101)
ABC294640 and doxorubicin	Preclinical	NSCLC (102)
ABC294640 and ABT-199	Preclinical	Multiple mieloma (103)
ABC294640 and regorafenib	Preclinical	HCC (104)
CNL	Preclinical	Breast cancer (105)
CNL	Preclinical	Chronic lymphocytic leukemia (106)
CNL	Preclinical	Melanoma and breast cancer (107)
CNL	Preclinical	Breast and pancreatic cancer (108)
CNL	Preclinical	Ovarian cancer (109)
CNL	Preclinical	Liver cancer (110)
CNL	Preclinical	Lymphocyte leukemia (111)
CNL	Preclinical	Melanoma (112)
CNL	Clinical (phase II)	Breast cancer (113)
CNL and sorafenib	Preclinical	Melanoma and breast cancer (114)
CNL, gemcitabine and PDMP	Preclinical	Pancreatic cancer (115)
CNL and tamoxifen	Preclinical	Triple-negative breast cancer (116)
CNL and vinblastine	Preclinical	HCC and colorectal cancer (117, 118)
CNL and tamoxifen	Preclinical	Colon cancer (119)
CNL and doxorubicin	Preclinical	Breast cancer and leukemia (120)
CNL and tamoxifen	Preclinical	AML (121)
CNL and chloroquine (CQ)	Preclinical	HNSCC (122)
CNL and PPMP	Preclinical	Leukemia (123)
CNL and vinblastine	Clinical (phase I), NCT02834611	AML or with MDS-related changes (AML-MRC) (118)
SKI-II	Preclinical	Gastric cancer (124)
SKI-II	Preclinical	Solid tumors (125)
SKI-II	Preclinical	Kidney adenocarcinoma (126)
SKI-II	Preclinical	HNSCC (127)
SKI-II	Preclinical	Breast cancer (128)
SKI-II	Preclinical	Prostate cancer (129)
SKI-II	Preclinical	Large granular lymphocyte leukemia (130)
SKI-II	Preclinical	Glioblastoma (131)
SKI-II	Preclinical	HCC (132)
SKI-II	Preclinical	HCC (133)
SKI-II	Preclinical	Colorectal cancer (134)
SKI-II	Preclinical	AML (135)
SKI-II	Preclinical	Gastric cancer, glioblastoma, cervical cancer (135)
SKI-II and myriocin	Preclinical	Merkel cell carcinoma (136)
SKI-II and temozolomide	Preclinical	Glioblastoma (137)
SKI-II and ABT-263	Preclinical	Leukemia (138)
SKI-II and gefitinib	Preclinical	Triple-negative breast cancer (139)
SKI-II and OSI-906	Preclinical	Breast cancer (140)
SKI-II and cisplatin	Preclinical	Gastric cancer (141)
SKI-II and curcumin	Preclinical	Ovarian cancer (142)
SKI-II and paclitaxel	Preclinical	NSCLC (143)
SKI-II and U0126	Preclinical	HCC (144)
SKI-II and EX527	Preclinical	Leukemia (145)
SKI-II and 5-FU	Preclinical	HCC (146)
α-GalCer	Preclinical	Ovarian and breast cancer metastasis (147)
α-GalCer	Preclinical	Breast cancer and melanoma (148)
α-GalCer	Preclinical	(149)
α-GalCer	Preclinical	(150)
α-GalCer	Preclinical	(151)
α-GalCer	Preclinical	Colon cancer (152)
α-GalCer	Clinical (phase I)	NSCLC (153)
α-GalCer	Clinical (phase I)	Melanoma (154)
α-GalCer	Clinical (phase I)	Lung cancer (155)
α-GalCer	Clinical (phase I)	HNSCC (156)
α-GalCer	Clinical (phase I)	HNSCC (157)
α-GalCer	Clinical (phase I)	NSCLC (158)
α-GalCer	Clinical (phase I)	HNSCC (159)
α-GalCer	Clinical (phase I)	HNSCC (160)
α-GalCer	Clinical (phase I)	Advanced cancer (161)
α-GalCer	Clinical (phase I)	NSCLC (162)
α-GalCer	Clinical (phase I)	Metastasis (163)
α-GalCer	Clinical (phase I)	NSCLC (164)
α-GalCer	Clinical (phase I)	Head and neck cancer (157)
α-GalCer	Clinical (phase I)	Melanoma (165)
α-GalCer	Clinical (phase I)	Metastasis (166)
α-GalCer	Clinical (phase I)	Metastasis (167)
α-GalCer	Clinical (phase I-II)	NSCLC (168)
α-GalCer	Clinical (phase II)	HNSCC carcinoma (169)
α-GalCer	Clinical (phase II)	NSCLC (170)
α-GalCer	Clinical (phase II)	NSCLC (171)
α-GalCer and SLP (palmitoylated synthetic long peptides)	Preclinical	Dendritic cells (172)
α-GalCer and irradiation or MHC-binding peptides	Preclinical	Lymphoma (173)
α-GalCer and pioglitazone	Preclinical	Melanoma (174)
α-GalCer and lenalidomide	Clinical (phase II), NCT00698776	Myeloma (175)
Fingolimod	Preclinical	Prostate cancer (176)
Fingolimod	Preclinical	Breast cancer (177)
Fingolimod	Preclinical	Prostate cancer (178)
Fingolimod	Preclinical	Pancreatic cancer (179)
Fingolimod	Preclinical	Prostate cancer (180)
Fingolimod	Preclinical	Prostate cancer (181)
Fingolimod	Preclinical	Breast and prostate cancer (182)
Fingolimod	Preclinical	Ovarian cancer (183)
Fingolimod	Preclinical	Breast Cancer (184)
Fingolimod	Preclinical	Breast cancer (185)
Fingolimod	Preclinical	Thyroid cancer (186)
Fingolimod	Preclinical	Prostate cancer (187)
Fingolimod	Preclinical	Colorectal cancer (188)
Fingolimod and TRAIL	Preclinical	Renal cancer (189)
Fingolimod and rapamycin	Preclinical	Pancreatic cancer (190)
Fingolimod, sphingosine, ISP-I-55 (FTY720 derivative)	Preclinical	Breast and colon cancer (191)
Fingolimod and sunitinib malate	Preclinical	Breast cancer (192)
Fingolimod and cisplatin	Preclinical	Ovarian cancer (193)
Fingolimod and cetuximab	Preclinical	Colon cancer (194)
Fingolimod, doxorubicin, and etoposide	Preclinical	Colon cancer (195)
Fingolimod, 5-FU, SN-38, and oxaliplatin	Preclinical	Colorectal cancer (196)
Fingolimod and radiation	Preclinical	Breast cancer (197)
Fingolimod and TRAIL	Preclinical	Renal, breast, and colon cancer (198)
Fingolimod and SKI-II	Preclinical	Uterine cervical cancer (199)
Fingolimod and docetaxel	Preclinical	Resistant prostate cancer (200)
Fingolimod and doxorubicin	Preclinical	Breast cancer (201)
Fingolimod and TASP0277308	Preclinical	Cancer-induced bone pain (202)
Fingolimod and cisplatin	Preclinical	NSCLC (203)
Fingolimod, carboplatin and tamoxifen	Preclinical	Ovarian cancer (204)
Fingolimod and methotrexate	Preclinical	Oral squamous cell carcinoma (205)
Fingolimod and gemcitabine	Preclinical	Pancreatic cancer (206)
Fingolimod and cisplatin	Preclinical	Breast cancer (207)
Fingolimod and pemetrexed	Preclinical	NSCLC and ovarian cancer cells (208)
Fingolimod, lapatinib and sorafenib	Preclinical	NSCLC (209)
Fingolimod and methotrexate	Preclinical	Thyroid cancer (210)
Fingolimod nanoparticles	Preclinical	Thyroid cancer (211)
Fingolimod	Preclinical	Breast cancer (212)
Sonepcizumab	Preclinical	Breast and ovarian cancer (213)
Sonepcizumab	Clinical (phase II)	Metastatic renal cell carcinoma (214)

AML, acute myeloid leukemia; CLL, chronic lymphocytic leukemia; HCC, hepatocellular carcinoma; HNSCC, head and neck squamous cell carcinoma; NSCLC, non-small cell lung cancer.

## Fenretinide

Fenretinide (N-(4-hydroxyphenyl)retinamide; 4-HPR) reduces the *de novo* synthesis of ceramide by targeting dihydroceramide desaturase (DES) while inducing an increase in dihydroceramide levels. This enzyme is responsible for the desaturation of dihydroceramide, the final step in the *de novo* synthesis of ceramide lipid species from dihydroceramide precursors. Dihydroceramides induce autophagy and inhibit cell growth by inducing cell cycle arrest in cancer cells (215, 216). In addition to DESs, other enzymes are fenretinide targets (i.e., CerS5).

Fenretinide treatment induces cell death through the following mechanisms: apoptosis (increased cleavage of caspases and PARP proteins; induction of NR4A1 expression, which interacts with Bcl-2, exposing aBH3 domain and a pro-apoptotic function; and induction of ATF3 expression, ATF4 expression, and NOXA transcription) (9, 11–14, 16, 18, 43); autophagy (increased LC3-II levels) (9); endoplasmic reticulum stress and accumulation of reactive oxygen species (ROS) (11, 14, 16–18, 43); repression of mammalian target of rapamycin (mTOR) signaling and a subsequent reduction in Erk1/2 activity (9); ceramide production (9, 17); antitumor activity against CSCs (reduced CD44, ALDH, Nanog, Sox2, and POU5F1 expression) (10, 16, 18); induction of cell cycle arrest (decreased p-AURA, CDC25, cyclin E2, and cyclin A2 levels and increased p16 levels) (9, 11, 12, 18); and p38-MAPK signaling (19) (**Figure 3**). Several articles have shown that fenretinide preferentially targets CSCs when sphere formation and stemness markers are analyzed (9–11, 14–16).

Preclinical studies have indicated the antitumor activity of fenretinide *in vitro* and *in vivo* in several tumor types in the absence of toxicity in mice. However, clinical trials have reported some mild side effects of fenretinide, such as musculoskeletal complaints (55), diarrhea, reversible night blindness, allergic reaction (21), and dermatological disorders (40). Furthermore, fenretinide lacks antitumor activity in most studies (n=13) but has been shown to stabilize the disease or exert protective effects on some cancers (n=6), mostly breast cancer. Fenretinide preferentially accumulates in fatty tissues, such as the breast, which may contribute to its effectiveness against breast cancer (42). Fenretinide has shown a lack of activity against other cancers. For example, fenretinide does not reduce the time to recurrence of renal carcinoma, consistent with low intratumor drug concentrations (33). Additionally, fenretinide does not substantially modulate the levels of several biomarkers in prostate cancer, including transforming growth factor alpha (TGF-α), insulin-like growth factor 1 (IGF-I), insulin-like growth factor binding protein 3 (IGFBP-3), sex hormone binding globulin (SHBG), and prostate-specific antigen (PSA), which are indicative of insufficient biological activity (36, 37). The remarkable hydrophobicity of this drug may be one of the factors responsible for its lack of effectiveness in clinical trials. Better formulations, such as encapsulation into nanocarriers for oral administration, have been reported to be a feasible option to increase its activity (13, 217).

However, fenretinide induces a positive hormonal (47) and metabolic profile in premenopausal women (50) and exerts a beneficial effect on total serum cholesterol and HDL levels (53). These beneficial effects have been observed in some cancers, such as breast cancer, but not in others, thereby indicating a possible specificity of fenretinide for this tumor type. Interestingly, there are some correlations between oncogenic alterations and the efficacy of this drug. For example, the sensitivity of Ewing’s sarcoma cells to fenretinide-induced cell death is decreased following downregulation of the oncogenic fusion protein EWS-Fli1 and p38(MAPK) activity (218). Also, fenretinide caused induction of oncogene c-Fos expression, whereas such an effect was not observed in resistant cells to fenretinide-induced apoptosis (219).

Also, the combination of fenretidine and ABT-263 (Bcl-2 inhibitor) induces the apoptosis of a large number of HNSCC cells, regardless of the human papillomavirus (HPV) or p53 status. The primary targets of apoptosis induced by these drugs are MCL1 (a Bcl-2 family apoptosis regulator), and Bcl-2 like 1 (Bcl-X_L_) (220). Remarkably, the nanomicellar combination of lenalidomide–fenretinide suppresses tumor growth in a MYCN-amplified neuroblastoma tumor mediated by increased expression of GD2, a disialoganglioside expressed on tumors of neuroectodermal origin (221). Moreover, treatment with a combination of fenretinide, tocilizumab, and reparixin significantly suppresses IL-6 release, IL-8 release, stem cell gene expression, and invasion in CSC populations (222), which may be due to increased ceramide levels and decreased IL6 and CXCR1/2 levels.

## Safingol

Safingol [(2S, 3S)]-2-aminoctadecane-1,3-diol] is an inhibitor of SPHK1, PKCβ-I, PKCδ, PKCϵ, PI3K, and glucose uptake (223). Safingol also affects the balance of ceramide/dihydroceramide levels. The inhibitory effects on signaling, particularly on PKCϵ and PI3k, concomitant with the presence of ROS (67) synergize to induce apoptosis (decreased Bcl-2 levels and increased caspase cleavage) (59, 60, 62–65, 68) and/or autophagy (63, 67) (**Figure 3**). According to preclinical studies, the combination of safingol with conventional chemotherapy agents, such as doxorubicin (67), irinotecan (66), and mitomycin C (65), potentiates their effects, inducing apoptotic cell death and ROS production in different cell lines. Additionally, the administration of safingol in combination with bortezomib inhibits lung tumor growth and metastasis (through the modulation of NF-κB signaling) in orthotopic syngeneic mouse models (69). Unfortunately, hepatic toxicity, renal toxicity, changes in liver histology, and decreases in body weight have been observed in mice treated with safingol (56, 57). Two out of two clinical trials have indicated stable disease or minor responses to safingol in a subgroup of patients (73, 74). However, hepatic toxicity has been observed in a clinical trial of safingol (73), resulting in few additional clinical trials of this drug. In resistant cancer types, such as gastroesophageal cancer, treatment with the combination of safingol with other chemotherapeutic agents, such as cisplatin, has been proposed to potentially overcome cytotoxic drug resistance. This conclusion is based on the following observations: i) cisplatin resistance correlates with increased SPHK1 expression and with decreased sphingosine-1-phosphate lyase 1 (SGPL1) expression; and ii) the survival of patients treated with chemotherapy prior to surgery but not patients treated with surgery alone (70).

## ABC294640

ABC294640 (opaganib, Yeliva, 3-(4-chlorophenyl)-N-(pyridin-4-ylmethyl)adamantane-1-carboxamide) is a selective inhibitor of both SPHK2 and DES1 that decreases the synthesis of the pro-proliferative and antiapoptotic lipid S1P, which may eventually lead to the induction of apoptosis and inhibition of cell proliferation in cancer cells overexpressing SPHK2 (75–77, 82). *In vitro* studies have indicated that ABC294640 reduces the proliferation and viability of several cancer cell lines and mouse xenografts without any toxic side effects. The decrease in proliferation is mediated by inhibition of SPHK2 activity (82, 85, 97), S1P depletion (76, 79, 84, 85, 95, 97, 99), accumulation of ceramide (79, 84, 85, 89, 99), induction of apoptosis (increased caspase cleavage, decreased Bcl-2 levels, and decreased NOXA transcription) (76, 78, 79, 84–91, 94–96, 98, 99, 102, 103), induction of autophagy (increased LC3-II and beclin-1 levels) (77, 83, 98), estrogen/androgen receptor signaling (decrease in progesterone or androgen receptor levels) (75, 81, 82), cell cycle arrest (increased Myt1, p-cdc2, p53, and p21 levels and decreased pRb, cyclin B1, and cyclin D1 levels) (81, 85–87, 100, 101), and modulation of cell survival pathways (decrease or inhibition of NF-κB, pERK1/2, pJNK, pAKT, c-Myc, and survivin expression, as well as p21-activated kinase 1 (PAK1)/p-Lin-11/Isl-1/Mec-3 kinase 1 (LIMK1)/Cofilin1 signaling) (77, 78, 81, 82, 84, 87, 90–92, 100–103) (**Figure 3**). The combination of ABC294640 with other drugs, such as regorafenib, sorafenib, PDMP, and ABT-199, induces synergistic potentiation of the treatment effect, reducing chemoresistance in various cancer types (98, 99, 103, 104). For example, SPHK2/SPP1 arbitrates regorafenib resistance by activating signal transducer and activator of transcription 3 (STAT3) and nuclear factor kappa light chain enhancer of activated B cells (NF-κB). SPHK2 targeting by ABC294640 significantly reduces resistance to regorafenib in an *in vivo* model of hepatocellular carcinoma (HCC) (104).

Overall, only one clinical trial for ABC294640 has been reported, and some reversible toxicities (nausea, vomiting, diarrhea, fatigue and nervous system disorders) were documented. These side effects are likely due to off-target effects. The efficacy evaluation indicated stable disease in a subgroup of patients (40%), partial response (7%) and progressive disease (53%) (93).

## Ceramide Nanoliposomes

Ceramide nanoliposomes (CNLs) are lipid-based nanoparticle formulations composed of ceramide encapsulated within nanoliposomes, inducing apoptosis in the target cells due to lysosomal membrane permeabilization that leads to the leakage of hydrolytic enzymes into the cytoplasm or by conferring PI3K and PKCζ tumor-suppressive activities (107, 224). Interestingly, CNLs have also been reported to target the Warburg effect in chronic lymphocytic leukemia *in vitro* and *in vivo* (106). Ceramide alone is insoluble and has a short half-life; therefore, nanoliposomes increase its solubility and half-life. Upon administration, CNLs accumulate in the tumor environment due to enhanced permeation and retention caused by the ‘leakiness’ of the tumor vasculature (225). No targeting effect on a tumor marker or tropism of CNL for a particular tissue has been observed. However, one method for increasing the specificity of ceramide derivatives for mitochondria (to induce apoptosis by inducing cytochrome c release) is the introduction of a positive charge on the fatty acid residue by adding a pyridine structure. Pyridine-ceramides localized more readily to the mitochondria, altering their structures and functions and inducing pancreatic cancer cell death (226).

Preclinical assays with cell lines and xenografts show that CNLs potentiate the effect of chemotherapy (114–116, 120); reduce tumor proliferation mediated by apoptosis (increased cleavage of PARP and caspases) (110–112, 114, 116–119, 121, 123), autophagy (increased LC3-II and Atg5 levels) (117, 122), necrosis (106), necroptosis (109), anoikis (108), mitophagy (mitochondrial membrane permeabilization) (116, 119, 121–123), and cell cycle arrest (increased p53 expression) (116, 119); increase ROS levels (110); inhibit lysosomal function (116, 122); inhibit integrin affinity (105, 107); and target CD44 receptor (108), survivin (111), PI3K (107, 114), MAPK (105, 114), mammalian target of rapamycin (mTOR) (112, 121), Akt and Erk1/2 (110, 115) signaling (**Figure 3**). For example, Shaw et al. indicated that the combination of C6-CNLs with chloroquine (an inhibitor of lysosomal function and therefore an autophagy inhibitor) significantly increases apoptosis in response to ceramide by avoiding the repair of mitochondrial damage (122).

To our knowledge, two clinical trials have tested the efficacy of CNLs in cancer. In the first trial, only one patient with cutaneous breast cancer manifested a partial response, yielding a response rate of 4% and a median progression-free survival of 2 months. Topical ceramides were also well tolerated, with no grade 3 or 4 toxicities reported (113). Another clinical trial (phase I) with C6-CNLs concluded that the combination of ceramide and vinblastine is safe and has the potential to treat the heterogeneous nature of acute myelogenous leukemia (AML) through the induction of apoptotic pathways (118); therefore, phase II studies may be conducted.

## SKI-II

SKI-II (SKi, SphK-I2, 4-[[4-(4-chlorophenyl)-1,3-thiazol-2-yl]amino]phenol) is a highly selective inhibitor of both SPHK1 and SPHK2 (227). *In vitro* studies have shown that SKI-II decreases cancer cell proliferation by inducing apoptosis (increased PARP cleavage, increased caspase cleavage, decreased Bcl-2 expression, and increased Bax levels) (124, 126, 129, 130, 135–137, 142, 144–146), autophagy (137), necrosis (136), endoplasmic reticulum stress, oxidative stress, and cell cycle arrest [increased levels of p27 and sirtuin-1 (SIRT1)] (124, 130, 145). In addition, SKI-II has been shown to decrease sphingomyelin and S1P levels (130, 136), inhibit chemotaxis (131), increase ceramide levels (126, 130, 137, 138, 142), and/or increase the activation of other crucial signaling pathways, including transcription factor NF-κB (124, 126, 144, 146), the Janus kinase 1-signal transducer and activator of transcription 1 axis (JAK-STAT) (130, 134, 145), mitogen-activated protein kinase 1 (MAPK) (125, 141, 146), Akt (125, 142–144, 228), Erk1/2 (141, 143–145), c-Jun NH2-terminal kinase 1 (JNK1) (191), tripartite motif containing 14 (TRIM14), metalloproteinases (MMP2 and MMP9), vascular endothelial growth factor (VEGF) (134), estrogens (128), Wnt family member 5A (Wnt5A) concomitant with β-catenin (132), epidermal growth factor receptor (EGFR), insulin-like growth factor binding protein 3 (IGFBP-3) (139), focal adhesion kinase (FAK), and insulin-like growth factor 1 receptor (IGF-1R) (146) (**Figure 3**). Sensitization of cell lines to SKI-II along with chemotherapy has also been observed (139, 141, 143). Unfortunately, clinical trials of this drug have not been conducted.

## α-Galactosylceramide (α-GalCer)

The last decade has revolutionized cancer therapy with the development of immunotherapy, producing good outcomes in patients with a fatal diagnosis. α-GalCer (KRN-7000, α-galactosylceramide-pulsed antigen presenting cells) is a glycosphingolipid and synthetic iNKT (invariant Natural Killer T) cell ligand. Dendritic cells are pulsed with α-GalCer and administered to patients for achieving effective presentation and activation to iNKT cells (172). In other approaches, dendritic cells are mixed with iNKT cells or peptides derived from cancer antigens (154). Dendritic cells (DC) capture antigens and present them to several types of T-cells for their activation. Invariant natural killer T (iNKT/type I NKT) cells are a subset of T cells endowed with innate and adaptive effector functions. They are characterized by the expression of invariant T cell receptor chain Vα24-Jα18, which recognizes lipid antigens presented by CD1d (229). They exhibit powerful cytotoxic activity mediated by perforin/granzyme B. In addition to their direct antitumor effect, iNKT cells also regulate the damaging activities of NK cells, CD8+ T cells, B cells and innate cells by release of a wide variety of pro-inflammatory cytokines (153, 154, 172).

Preclinical and clinical trials using α-GalCer have shown that this therapy is safe, exhibits durable activation, and increases the number of iNKT, NK, tumor-specific, CD4+, CD8+ T, and B cells (148, 149, 151, 153–156, 160, 161, 169, 172, 173, 175). This activation is associated with increased serum levels of cytokines that stimulate the growth and function of T cells [IL-12 (150, 175) and IL-2 receptors (175)] and other factors that enhance natural killer cell activity (i.e., interferon gamma [IFN-γ] (150, 155, 156, 158, 161, 163, 172), CD16 (175), and tumor necrosis factor α [TNF-α]) and immune cell maturation (GMCSF) (164). In eleven out of twelve completed phase I-II clinical trials, tumor regression, stable disease, partial response or increased median survival time were observed in a subgroup of patients (153, 157, 159, 160, 162–169). These promising clinical findings are associated with the activation of natural killer cells, cytotoxic CD8+ T cells and CD4+ T cells, which are the most relevant immune responses to cancer (230).

Attempts to improve efficacy of iNKT treatments have focused on transduced with CARs (chimeric antigen receptors) (NCT03294954; NCT03774654), chemical modifications to the α-GalCer to stabilize interactions with CD1d, optimizing presentation through encapsulation in particulate vectors, making structural changes that help binding to CD1d, injecting agonists covalently attached to recombinant CD1d. Also, facilitate formation of resident memory CD8+ T cells could find a role in this therapy.

## Fingolimod

Fingolimod (FTY720, Gilenya, 2-amino-2-[2-(4-octylphenyl)ethyl]propane-1,3-diol) is a functional antagonist of the sphingosine-1-phosphate receptor (S1PR) and structural analog of sphingosine (1). Fingolimod causes the internalization of S1PR, which sequesters T lymphocytes in lymph nodes (absent in the periphery) (231), preventing them from contributing to inflammatory and autoimmune reactions. The most universal mechanism for its potential anticancer function is limiting the conversion of sphingosine to S1P (7). Fingolimod is effective at reducing inflammatory relapses in patients with multiple sclerosis (232). Fingolimod also shifts macrophages to an anti-inflammatory M2 phenotype and modulates their proliferation, morphology, and cytokine release (233). Preclinical studies of fingolimod have indicated that this drug is safe, potentiates the effect of chemotherapy (192, 195, 196, 200, 201, 234), and suppresses tumor growth by inducing apoptosis (increased cleavage of PARP and caspases, decreased Bcl-2 and Mcl-1 levels, and increased Bax levels) (176, 177, 179, 181, 182, 187, 189, 194–199, 203, 204, 206), autophagy (increased LC3-II, beclin-1, and Atg7 levels, and decreased p62 expression) (197, 203, 208, 209), necrosis (183, 210), cell cycle arrest (increased levels of cell cycle inhibitory proteins [p27 and p21]); and decreased expression of cyclin D1 and C-X-C motif chemokine receptor 4 [CXCR4]) (186, 187, 197, 204, 209). Fingolimod also increases ceramide levels (181, 204), the proteasomal degradation of SPHK1 (182), inactivation of RhoA-GTPase (178), histone deacetylase (HDAC) activity (185), multidrug resistance protein 1 (ABCB1) levels (195), protein phosphatase 2A (PP2A) reactivation (196, 205, 206), and modulation of signaling pathways (VEGF (176, 186, 199), MMP2, MMP9, CD31, E-cadherin, β-catenin (176), estrogens (187), JNK (191), NF-κB (206), STAT3 (201, 206), AMP-activated protein kinase (AMPK) (208), mTOR (208), Erk1/2 (182, 186, 189, 191, 196, 197, 206), and PI3K/Akt (179, 180, 194, 196, 197, 206) (**Figure 3**).

However, no clinical trials have assessed the effectiveness of fingolimod in cancer, potentially due to the impairment of cytotoxic CD8+ T and CD4+ T cell trafficking and activation, which precludes tumor infiltration to kill cancer cells. Fingolimod blocks the immunosurveillance of B cells by suppressing the migration of tumor-specific Th1 cells from lymph nodes to the incipient tumor site, thereby preventing Th1-mediated activation of tumoricidal macrophages (235). Furthermore, it impairs the ability of cytotoxic CD8+ T cells to kill their target cells and reduces IFNγ and Granzyme B levels in splenic CD8+ T cells (236, 237). Thus, an effective action of this drug in clinical trials is not anticipated, as T cells are the main cells involved in the immune response to tumors.

## Sonepcizumab

Sonepcizumab (LT1009) is a humanized monoclonal antibody against S1P. Sonepcizumab slows tumor progression in murine models with orthotopic tumors by blocking the function of proangiogenic growth factors (decreased VEGF, bFGF, and IL-8 levels) and inducing apoptosis (increased caspase cleavage). Additionally, sonepcizumab inhibits tumor vascularization *in vitro* and *in vivo*, and it neutralizes S1P-induced stimulation of proliferation in multiple cell lines (213) (**Figure 3**). A phase II study of sonepcizumab was terminated because it failed to meet its primary progression-free survival endpoint in patients with metastatic renal cell carcinoma who received three prior therapies. However, researchers were encouraged by the overall survival (21.7 months) and safety profile of sonepcizumab, and they advised “further investigation in combination with VEGF-directed agents or checkpoint inhibitors”. Ten percent of patients achieved a partial response, with a median duration of response of 5.9 months. No grade 3/4 treatment-related adverse events were observed in >5% of patients (214).

An increase in systemic S1P concentrations was detected following sonepcizumab treatment, suggesting that S1P signaling was still active, which might explain the limited efficacy of the drug in the clinic. Thus, future studies are needed to improve the neutralization of S1P signaling. In addition, studies testing the efficacy of this drug in combination with SPHK1/2 inhibitors or S1PR2 antagonists are warranted (1).

## Conclusions

Sphingolipid-targeting drugs have been tested against several hematological malignancies and solid tumors, alone or in combination with chemotherapy, and have produced some encouraging results (42, 47, 48, 50, 52, 54). Treatments targeting sphingolipid exhibit antitumor activity *in vitro* and *in vivo*, inducing apoptosis or occasionally autophagy, as well as several other mechanisms of cell death. Among these agents, the most effective and promising treatments in clinical trials are fenretinide and α-galactosylceramide. Some plausible explanations for the partial success of these safe drugs in clinical trials have been proposed. Fenretinide accumulation in breast tissue along with the induction of apoptosis or autophagy (in caspase-defective breast cancer cells) by dihydroceramide may be responsible for its success. Researchers presumed that its accumulation in breast tissue (and not in other tissues) might be related to hormone-associated pathways that are active in these cancer types. Regarding α-galactosylceramide, the induction of an antitumor immune response mediated by iNKT, NK, T cells and B cells is the functional mechanism. Among several anticancer therapies, immune checkpoint inhibitors occupy a relevant place because of the activation of the antitumor function of T cells (238), which indirectly indicates an important role for the adaptive immune system in the efficacy of anticancer treatments. However, despite different proposals (mutations that prevent T cells from entering the tumor, inhibition of T cell activation pathways, etc.), researchers have not yet clearly determined why immunotherapy is not efficient against some types of tumors.

Current research gaps in the other drugs are associated with side effects, modest findings or the absence of clinical trials. For example, safingol and ABC294640 induced side effects on humans in clinical trials, which may be the main reason for the limited number of clinical trials. Safingol is an inhibitor of several enzymes (SPHK1, PKCβ-I, PKCδ, PKCϵ, and PI3K) and glucose uptake (223), which are needed for the proper function of normal tissues. Targeted therapy against protein kinases relies on the upregulation/activation of these molecules in particular tumors. For example, imatinib is a specific inhibitor of the constitutively active Bcr-Abl tyrosine kinase and is used to treat leukemia with the Philadelphia chromosome (Bcr-Abl) (239). Therefore, we understand that off-target effects of sanfingol due to the inhibition of several enzymes and glucose uptake are likely responsible for the hepatic toxicity observed in mouse and human studies. Potential developments in this field to alleviate this limitation might include some chemical modifications designed to increase the specificity for SPHK1 or targeting an upregulated sphingolipid in a specific tumor. Nevertheless, their use is expected to vary depending on the type of cancer, which in turn is determined by the levels of aberrant sphingolipids expressed in each type of tissue, among other factors. In addition, glucose uptake is a universal and vital step for obtaining ATP through glycolysis and oxidative phosphorylation.

CNLs are already being investigated in clinical trials, but the expected results were very modest, potentially because of a lack of CNL tropism for a specific tumor tissue type (i.e., breast). No clinical trials for SKI-II and fingolimod have been reported. For the latter, an effective action in cancer clinical trials is not expected, as this immunosuppressive drug impairs the tissue infiltration and activation of cytotoxic CD8+ T and CD4+ cells, which are the most relevant cells involved in the immune response to tumors. Clinical studies confirm this fact, as spontaneous regression of T cell lymphoma has been observed in patients with multiple sclerosis after discontinuing fingolimod (240).

With respect to sonepcizumab, an increase in systemic S1P concentrations was observed in a clinical trial (214), although it is a monoclonal antibody against S1P. Treatment with this drug resulted in a reduction in the absolute serum lymphocyte levels, which was expected based on the known effect of S1P blockade on peripheral lymphocyte trafficking (214). Moreover, upregulation of the S1PR1-STAT3 pathway enables myeloid cells to intravasate and mediate tumor proliferation and metastasis (241). In addition, S1PR1 signaling in T cells drives Treg accumulation in tumors, limits CD8+ T cell recruitment and activation, and promotes tumor growth (242, 243). Therefore, sonepcizumab does not provide effective S1P blockade in clinical trials, and the potential tumor infiltration of Tregs and myeloid cells and reduction of lymphocyte numbers fosters tumor growth.

The exhaustive characterization at several levels, including immunity, pharmacodynamics, pharmacokinetics, dosing and metabolomics, is required in preclinical studies before entering clinical trials. The most relevant factor associated with side effects is the presence of off-target effects, which might be improved by chemical modification of these drugs or new synthesis to increase specificity. For this task, the use of molecular docking based on three-dimensional protein structures would be able to develop new and more specific drugs. In addition, the lack of tissue-specific targeting and hydrophobicity of the drugs precludes an effective action. The use of aberrant sphingolipids in specific tumors as targets and nanocarriers or chemical modifications are solutions to these issues.

Aberrant sphingolipid signaling is a consequence (not the cause) of carcinogenesis due to mutations in crucial oncogenes and tumor suppressor genes. Hence, effective treatment with sphingolipid modulating drugs should be based on multiple therapeutic combinations, including immunotherapy (activates antitumor immune CD4+ and CD8+ T cells) and conventional chemotherapy Interestingly, conventional chemotherapy (i.e., tamoxifen) is active against SPHK1 and GCS; thus, the use of tamoxifen might be beneficial in patients who have acquired resistance to these enzymes. One opportunity is based on the fact that many chemotherapeutic agents modulate ceramide levels; therefore, the rational use of these agents with sphingolipid inhibitors could increase lethal levels of ceramide that are more effective at killing the tumor. Overall, an increased understanding of the mechanisms by which sphingolipids control cancer cell signaling together with in-depth studies using animal models will fill these gaps and improve future anticancer therapy based on these compounds.

## Author Contributions

OC write the manuscript. CM designed the figures accordingly to the literature. YG-M revised the manuscript. ML revised and re-write the manuscript. All authors contributed to the article and approved the submitted version.

## Funding

This work was supported by grants from the Instituto de Salud Carlos III (ISCIII; PI20/00556 and CP03/00101 [ML]) and CIBERONC (ML). This work was also co-financed by the European Regional Fund (ERDF) and AECC (Spanish Association of Cancer Research) (Founding Ref. GC16173720CARR [ML]). YG-M and CM were supported by the VHIR and iP-FIS (ISCIII) fellowships, respectively.

## Conflict of Interest

The authors declare that the research was conducted in the absence of any commercial or financial relationships that could be construed as a potential conflict of interest.

## Publisher’s Note

All claims expressed in this article are solely those of the authors and do not necessarily represent those of their affiliated organizations, or those of the publisher, the editors and the reviewers. Any product that may be evaluated in this article, or claim that may be made by its manufacturer, is not guaranteed or endorsed by the publisher.
